# Postinfarct Left Ventricular Remodelling: A Prevailing Cause of Heart Failure

**DOI:** 10.1155/2016/2579832

**Published:** 2016-02-18

**Authors:** Alessio Galli, Federico Lombardi

**Affiliations:** Cardiovascular Diseases Unit, Fondazione IRCCS Ca' Granda Ospedale Maggiore Policlinico, Department of Clinical and Community Sciences, University of Milan, Via F. Sforza 35, 20122 Milan, Italy

## Abstract

Heart failure is a chronic disease with high morbidity and mortality, which represents a growing challenge in medicine. A major risk factor for heart failure with reduced ejection fraction is a history of myocardial infarction. The expansion of a large infarct scar and subsequent regional ventricular dilatation can cause postinfarct remodelling, leading to significant enlargement of the left ventricular chamber. It has a negative prognostic value, because it precedes the clinical manifestations of heart failure. The characteristics of the infarcted myocardium predicting postinfarct remodelling can be studied with cardiac magnetic resonance and experimental imaging modalities such as diffusion tensor imaging can identify the changes in the architecture of myocardial fibers. This review discusses all the aspects related to postinfarct left ventricular remodelling: definition, pathogenesis, diagnosis, consequences, and available therapies, together with experimental interventions that show promising results against postinfarct remodelling and heart failure.

## 1. Introduction

The number of persons surviving an acute coronary syndrome has increased in the last decade [1], as a consequence of several improvements in the care of patients: more effective therapies, the development of a network of emergency intervention, door-to-balloon time of 90 minutes or less in the growing number of hospitals equipped to perform a primary percutaneous coronary intervention (PPCI) and better understanding of alarm symptoms of coronary heart disease among people [2–5]. All these elements contribute to reduce the loss of viable myocardial tissue in patients with myocardial infarction. However, in spite of a significant reduction in short-term mortality in patients with myocardial infarction, it has been observed an increase in long-term morbidity due to chronic heart disease, as shown by the statistics of hospital discharges for heart failure in the United States in the last 30 years (from about 440.000 in 1980 to 1.023.000 in 2010) [1].

In the United States, it is estimated that about 860.000 persons survive a first or recurrent heart attack every year [1], leading to an equal increase in the number of patients at risk of developing heart failure, the disease with the highest social and economic cost in western countries (57.757 deaths with $31 billion per year in the United States) [1]. Heart failure is a chronic and progressive disease characterized by a symptomatic impairment in cardiac function [6]. 5.1 million adult Americans live with it, and it is estimated that by 2030 more than 8 million adults living in the United States will have heart failure. Mortality is high, reaching 50% at 5 years from diagnosis of heart failure [1]. Heart failure is divided into two categories: heart failure with reduced ejection fraction (HFrEF), accounting for 50 to 70% of cases, and heart failure with preserved ejection fraction (HFpEF). A left ventricular ejection fraction (LVEF) ≥ 50% discriminates HFpEF from HFrEF, indicating a diastolic dysfunction more than systolic dysfunction [7].

The American Heart Association and the American College of Cardiology jointly released a classification of chronic heart failure based on four stages, with disease severity increasing from the first to the fourth stage [8]. Stage A is the presence of risk factors for heart failure without structural heart disease, stage B is the presence of a structural heart disease without symptoms, stage C is symptomatic heart failure, and stage D is symptomatic heart failure that is refractory to medical therapy. Structural heart diseases include ventricular hypertrophy in hypertensive patients, valvular diseases, cardiomyopathies, and scars due to previous myocardial infarctions [8]. Notwithstanding a reduction of about 50% of infarct size with modern revascularization strategies as compared with no reperfusion [9], heart failure develops within 5 years of a first myocardial infarction in 8% of men and 18% of women between 45 and 64 years of age [1]. As the incidence of heart failure increases with age, it is likely that the higher incidence in women is due to an older age at the time of first myocardial infarction [1]. Animal models of myocardial infarction and cardiac imaging on patients with ischemic cardiomyopathy revealed that heart failure is preceded by an increase in ventricular volumes [10, 11]. This process has been termed postinfarct ventricular remodelling, and it implies an enlargement of left ventricular chamber, which passes from an elliptical to a more spherical shape [10]. This change is described by an increase in sphericity index [12], that is, the ratio between the actual left ventricular volume and the volume of a sphere whose diameter is equal to the major axis of the left ventricle. Normal values for sphericity index are 0.29 ± 0.7 at end diastole and 0.15 ± 0.8 at end systole [13].

It is known that chronic *β* adrenergic stimulation and renin-angiotensin system activation promote postinfarct remodelling, and long-term use of drugs that inhibit these two pathways is nowadays the best strategy to prevent heart failure in patients with a history of myocardial infarction [6, 8].

The knowledge of mechanical and molecular factors leading to ventricular remodelling could guide the development of new targeted therapies against heart failure.

## 2. Definition and Pathogenesis

### 2.1. Definition and Diagnosis of Postinfarct Ventricular Remodelling

Postinfarct ventricular remodelling develops in about 30% patients with a history of myocardial infarction [14]. As remodelling depends on infarct size [15, 16], it is likely that its prevalence is higher in the subgroup of patients without any or successful reperfusion. In a recent survey on patients admitted to 80% of the intensive coronary care units of Italian hospitals, only 60% of patients with ST-elevation acute coronary syndromes could be treated with reperfusion [17].

Ventricular remodelling is a predictor of heart failure, and for this reason it assumes a negative prognostic value [10].

An arbitrary definition of ventricular remodelling, but widely adopted in follow-up studies [18, 19], is an increase of at least 20% of left ventricular end-diastolic ventricular volume (LVEDV) from the first postinfarction imaging. However, as the first imaging study with cardiac magnetic resonance is usually performed a few days after myocardial infarction, early ventricular remodelling, which is the phase of remodelling that occurs in the first hours after myocardial infarction, could not be recognized, leading to an underestimation of the final ventricular dilatation [14].

Left ventricular remodelling is characterized by a progressive increase in both end-diastolic (LVEDV) and end-systolic volumes (LVESV). The increase in LVESV can precede the increase in LVEDV, as a consequence of an impaired systolic function that causes a reduction in stroke volume [14, 20].

The imaging modalities used to noninvasively assess ventricular volumes and function are echocardiography, radionuclide ventriculography, and cardiac magnetic resonance (CMR) [21]. In particular, cine CMR is the preferred method because it allows for a more accurate estimate of cardiac volumes, but it is not yet available in all hospitals [22, 23].

Ventricular volumes are best expressed as volume indices, which are obtained by dividing the volumes by the body surface area. Normal values for LVEDVI and LVESVI are 75 ± 20 mL/m^2^ and 25 ± 7 mL/m^2^, respectively, [24]. Volume indices allow for a reduction in interindividual variance that also depends on wider ventricular chambers in men than women [24, 25].

A reduction in left ventricular ejection fraction (LVEF) is often observed during postinfarct remodelling, predicting heart failure and increased mortality. Normal values of LVEF are 67 ± 8% [21] and depend on a preserved global systolic function [10, 26]. However, initial ventricular remodelling is not always associated with a reduction in LVEF, as this measure of systolic function can remain unchanged or even increase in the months following an acute myocardial infarction, even in the presence of an enlargement of ventricular chambers [27].

### 2.2. Pathogenesis

Ventricular remodelling accompanies different heart diseases, such as dilatative nonischemic cardiomyopathy and cardiac hypertrophy in chronic hypertension and implies a change in myocardial anatomical structure [28]. Postinfarct remodelling is a specific type of left ventricular remodelling that is a consequence of an increase in both preload and afterload causing an enlargement of ventricular chamber and a hypertrophy of normal myocardium [28]. The increase in preload is sustained by the phenomenon of infarct expansion, which is an enlargement of infarct scar [29]. This causes a regional increase in the ventricular volume subtended by the expanded infarcted myocardial wall.

**Figure 1:**
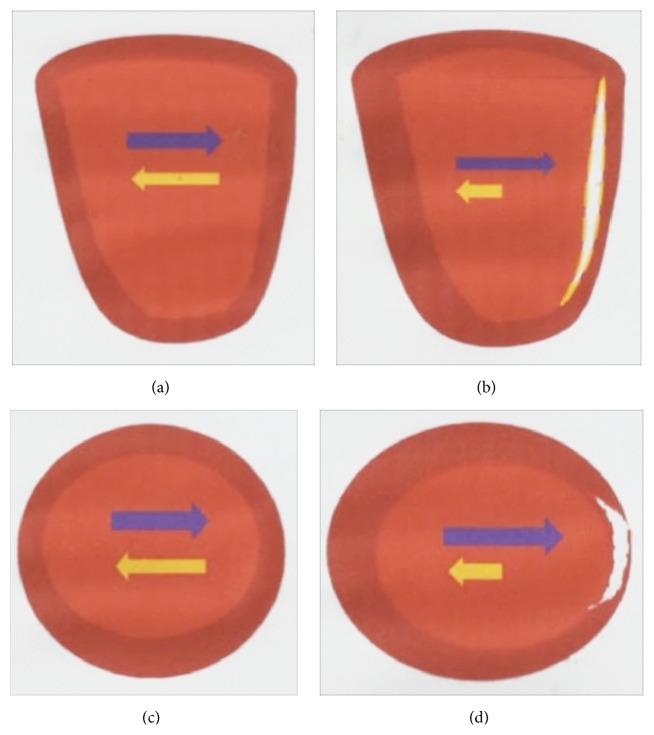
In a normal ventricle, the force generated by myocardial contraction is balanced (a and c). When there is an infarct scar (white), the infarcted segment is stretched by the force generated by the remote normal myocardium (b and d). As a result, the infarct scar expands and the infarcted wall becomes thinner, while the remote myocardium becomes hypertrophic to maintain a normal global cardiac function (d). Arrows indicate the vectors of forces generated by opposite left ventricular segments during systole.

In infarcted myocardium, ventricular contraction is not symmetrical, because the necrotic segments have lost their contractility [30]. As a result, the force generated by the normal remote myocardium during contraction is not counterbalanced by an equal and opposite force, and the infarcted ventricular wall is thus stretched by an increased wall tension that is not homogeneously distributed in the left ventricle [31] (Figure 1). This phenomenon might explain why the infarcted wall usually has longer contraction times than the healthy remote myocardium. In effect, the infarcted wall has to counteract a greater resultant force, and its prolonged time to peak systolic velocity can be detected as an asynchrony of ventricular wall motion [32]. This wall motion defect has been recognized as a risk factor for the development of remodelling, and it can be assessed with echocardiography or cine CMR [33, 34].

It is likely that some segments might recover a normal or near normal contractility in the months after myocardial infarction [30], because of the end of myocardial stunning [9, 35]. This is a reversible form of ischemia-reperfusion injury consisting in a dysfunction of myocardial tissue in the salvaged area at risk. As it is reversible, it is improbable that myocardial stunning contributes to ventricular remodelling. However, in transmural infarcts, some segments can remain hypokinetic, akinetic, or dyskinetic in areas where irreversible injury took place, causing a permanent regional ventricular dysfunction [30].

To maintain a normal stoke volume with a reduced number of normally working myocardial segments, the healthy myocardium has to produce a greater pressure [28]. The increase in workload (afterload) on healthy myocardium causes a hypertrophy of cardiomyocytes [28]. This phenomenon has been observed both in animal models of myocardial infarction [36] and more recently in men, using diffusion CMR tractography [37, 38]. Tractography with cardiac magnetic resonance has been recently introduced as a novel experimental imaging that allows for an in vivo study of the structure of the myocardial fibers that compose the ventricular wall [21, 39].

Diffusion CMR is capable of detecting the direction of H_2_O molecules diffusing in solution. Direction of myocardial fibers can thus be identified, because water mainly diffuses along the major axis of cardiomyocytes [21, 39].

The ventricular wall is composed of three layers of fibers with different orientation that rotate from the subepicardial to the subendocardial layer by almost 180° [21, 39].

The external layer is composed of left-handed helical fibers that constitute the anterior basal and the posterior apical portions of the left ventricle and encircle the ventricular chamber with an orientation between −90° and −30°, having its long axis as 0° [21, 39]. Subendocardial fibers have an opposite orientation, with a course from the posterior basal segments of the left ventricle to the anterior apical wall. Subendocardial fibers are right-handed helical fibers and make with the long axis of the ventricle an axis between +30° and +90° [21, 39]. Fibers in the midmyocardial wall, between the subepicardial and the subendocardial layers, are circumferential and are parallel to the short axis of the left ventricle [21, 39]. In noninfarcted heart, the thickness of fibers is similar between the three layers that compose the ventricular wall [21, 37–39].

After a myocardial infarction, diffusion CMR tractography evidences the disappearance of subendocardial fibers and a hypertrophy of the subepicardial layer in the infarcted segments [37–39]. However, the hypertrophy is not sufficient to prevent the thinning of infarcted ventricular wall [12]. The areas with no fibers correspond to infarct scar, where dead cardiomyocytes have been replaced by collagen [39]. Traditional CMR can identify with precision this area of irreversible myocardial injury, which appears as delayed hyperenhancement with gadolinium. Postinfarct remodelling is characterized by a structural change in myocardial fibers, which is present not only in ventricular segments directly damaged by myocardial infarction, but also in remote, apparently healthy, myocardial regions. In effect, in patients with a previous myocardial infarction, subendocardial fibers in the remote myocardium are hypertrophic [37, 38].

In postinfarct ventricular remodelling, hypertrophic cardiomyocytes are longer than normal cardiac cells. In an animal model, postinfarct ventricular remodelling was characterized by a lengthening of cardiomyocytes especially in the area surrounding the infarct scar, but also in remote myocardium [36]. This type of ventricular hypertrophy has been termed eccentric and contributes to the worsening of ventricular dilatation during remodelling. It is due to volume overload [28].

Cardiomyocytes modify their transcriptional activity during remodelling, reactivating the expression of fetal genes that are normally silenced during adult life [40]. These include genes encoding for structural heart proteins, which allow for the lengthening of cardiomyocytes through the addition of new sarcomeres in series [41]. Myofibrils undergo a qualitative alteration, because cardiomyocytes reduce the synthesis of isoform *α* of myosin heavy chain (*α*-MHC) to increase the production of isoform *β* (*β*-MHC) [40, 42]. This change is associated not only with a reduced energetic requirement to cardiac muscle, but also with a reduced contractility of sarcomeres [43]. The force generated by each contractile unit is further decreased by the reduction in the mean number of myofibrils per sarcomere [44].

HDAC inhibitors (HDACi) are a class of anticancer drugs designed to modulate gene expression in cancer cells [45]. In animal models, HDACi were also effective against pathologic cardiac hypertrophy [45, 46]. Treatment with HDACi blocked the fetal cardiac gene program that is activated in heart failure and increased the ratio of *α*-MHC to *β*-MHC [46]. The knowledge of the transcriptional changes associated with ventricular remodelling and heart failure might prompt the discovery of new drugs capable of modulating the expression of specific genes involved in the disease, possibly with limited untoward effects.

As heart has poor, if not absent, regenerative capacity, cardiac hypertrophy that occurs during postinfarct remodelling is accompanied by an increase in extracellular matrix, which is mainly constituted by collagen [47, 48]. This phenomenon is due to an increased activity of cardiac fibroblasts in response to different soluble fibrogenic mediators, such as transforming growth factor-*β* (TGF-*β*) and systemic and local activation of renin-angiotensin-aldosterone system (RAAS) [28]. The mediators of the RAAS that promote ventricular remodelling are angiotensin II and aldosterone [28].

It is probable that the increase in wall stress in the infarcted heart that becomes dilated during remodelling [31], as described by Laplace's law, accelerates collagen synthesis by cardiac fibroblasts [28]. The expansion of extracellular matrix reduces the stress on cardiomyocytes, but on the other hand it impairs ventricular function [28]. In effect, a negative correlation between extracellular matrix volume in remote myocardium quantified by contrast-enhanced CMR and left ventricular ejection fraction (LVEF) has been demonstrated [48]. Furthermore, the extent of interstitial myocardial fibrosis correlates positively with mortality [49]. An excess of extracellular matrix becomes maladaptive when diffusion of oxygen, fatty acids, and glucose from capillaries to cardiomyocytes is impaired by an increased extravascular space. Chronic deficit of oxygen can lead cardiomyocytes to apoptosis [50].

Remodelling is a pathologic process that involves the entire ventricle, leading to a change in its global structure [10, 28]. There are two types of causes of remodelling: mechanical and biochemical. While mechanical causes, as previously described, are an increase in both preload and afterload [28], biochemical causes are linked to the production of soluble mediators capable of promoting ventricular remodelling [28]. For example, angiotensin II and aldosterone stimulate cardiac hypertrophy and fibrosis, and an increase in catecholamines helps to maintain a normal cardiac output in front of the contractile dysfunction of infarcted segments [28, 44, 51]. Many other soluble factors are produced by cardiac cells in response to various types of potential damage, for example, ischemia-reperfusion injury [52, 53] or mechanical strain [54]. This explains the link between mechanical and biochemical causes of postinfarct remodelling.

Chronic volume overload and increased adrenergic tone promote metalloproteinases activity [55]. These proteolytic enzymes break down collagen cross-links, thus weakening myocardial wall and worsening ventricular chamber dilatation [28]. MMP-9 probably is the most important metalloproteinase involved in ventricular remodelling [56]. It has been suggested that collagen degradation during postinfarct remodelling is due to an imbalance between the activity of matrix metalloproteinases and tissue inhibitors of matrix metalloproteinases, in particular of TIMP-1 and TIMP-2 [57]. Increased plasma levels of MMPs, TIMPs, and collagen-derived peptides have been detected in patients with postinfarct ventricular remodelling, indicating an increased collagen turnover [56–58].

Cardiomyocytes become hypertrophic in response to integrin-mediated mechanotransduction [54] and soluble factors produced during myocardial stress [28]. Some of these ligands are growth factors with a protective role, promoting cell survival upon activation of tyrosine kinase receptors (Table 1) [28, 59]. Other ligands have a dual activity, with either adaptive or maladaptive roles, depending on concentration and duration of stress (Table 1) [28]. For example, angiotensin II can promote cell survival through the pathway of the extracellular-regulated kinase (ERK), but an excess of the angiotensin receptor activity during ventricular remodelling leads to the activation of the Jun N-terminal kinase (JNK) pathway and consequently to cardiomyocyte apoptosis [28]. Like angiotensin II, other mediators with dual activity have growth-promoting effects on cardiomyocytes at low doses, but they cause apoptosis at high concentrations or with chronic exposure [28].

## 3. Predictors of Remodelling

### 3.1. Predictors of Postinfarct Ventricular Remodelling

Ventricular remodelling usually develops in patients with a history of ST segment elevation myocardial infarction (STEMI), which produces an infarct scar with a transmural extent [15]. There are many predictors of ventricular remodelling that can be assessed with different cardiac imaging modalities. Patients who develop postinfarct left ventricular remodelling usually have a greater LVESV and a lower LVEF as postinfarct baseline characteristics [16]. The best independent predictor of left ventricular remodelling is infarct size, which can be quantified as the percentage of left ventricular mass with late gadolinium enhancement on CMR images [15, 83]. Anterior infarcts are usually larger, because the anterior interventricular branch of the left coronary artery is the most important arteriosus vessel to the heart.

During an acute myocardial infarction, plasma levels of cardiac troponins and creatine kinase-MB positively correlate with infarct size determined by CMR, and very high levels predict an increase in ventricular volumes and a reduction in LVEF [20, 84]. A10% increase in cardiac mortality for every 10% increment in infarct size has been estimated [85].

It is believed that heart failure develops when at least 25% of the left ventricular myocardial mass is lost [5]. The greatest number of cardiomyocytes dies during an acute myocardial infarction or during reperfusion, as a consequence of ischemia-reperfusion lethal injury [9, 35], but cardiomyocytes can also undergo apoptosis because of the chronic myocardial stress of postinfarct remodelling [86].

Other predictors of ventricular remodelling are the irreversible forms of ischemia-reperfusion injury of the cardiac microvasculature, which are microvascular obstruction (MVO) and intramyocardial hemorrhage (IMH) [87–90].

**Figure 2:**
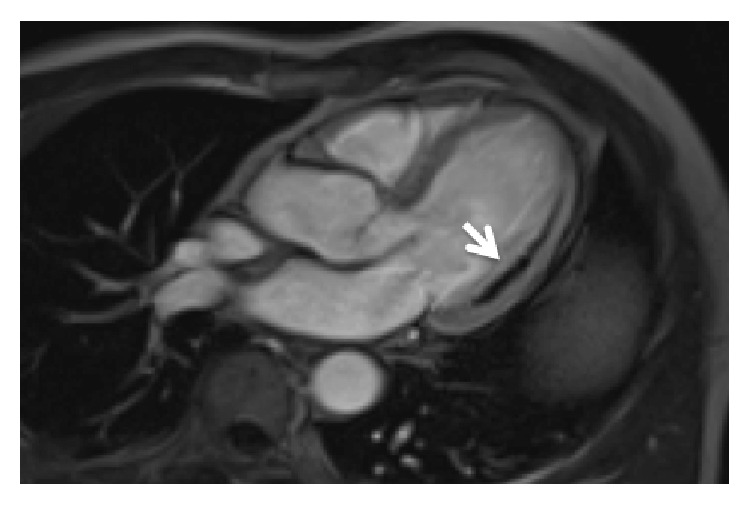
Microvascular obstruction (arrow) as shown by early gadolinium enhancement in a patient with acute myocardial infarction (CMR study).

MVO is identified as a hypointense area within the infarcted myocardium on CMR images of early and late gadolinium enhancement (Figure 2) [91–94]. This phenomenon, that is, the no-reflow of myocardial tissue, can be observed in up to 50% of patients with STEMI [94]. In many studies, it has been diagnosed as a low angiographic myocardial blush/perfusion grade, or a ST segment resolution <70% after primary percutaneous coronary intervention [92–94].

IMH is associated with a large infarct size and a large area of MVO [95]. Its presence and extension can be assessed with CMR using T2^*∗*^ mapping, because of the superparamagnetic properties of iron-containing hemoglobin degradation products that reduce myocardial signal in the hemorrhagic area [87, 95, 96]. The association between IMH and postinfarct remodelling might be explained by chronic inflammation and impaired healing of infarcted tissue [97], owing to the presence of a chronic iron deposit within the necrotic myocardium [98]. Free iron catalyzes the production of free radicals, which cause oxidative stress [99]. Furthermore, the intramyocardial hemorrhage seems to worsen the systolic dysfunction of infarcted segments, promoting infarct expansion and ventricular dilatation [100].

MVO and IMH are also independent predictors of major adverse cardiac events (MACE), including cardiac death, stroke, myocardial infarction, and hospitalization for heart failure: hazard ratio (HR) for MVO is 2.79 (95% CI: 1.25–6.25, *p* = 0.012) [101], and HR for IMH is 1.17 (95% CI: 1.03–1.33, *p* = 0.01) [89].

After a myocardial infarction, the most frequent of the posterior basal segments, a dysfunction of a papillary muscle can occur, leading to a mitral regurgitation [14, 28]. The blood volume of the regurgitation increases the ventricular preload, contributing to remodelling. For this reason, a clinically significant mitral regurgitation is a risk factor for postinfarct ventricular remodelling [28, 102]. The progressive enlargement of the ventricular chamber worsens further the function of the mitral valve, through the dilatation of the mitral annulus [14, 28].

The presence of an aortic stenosis or hypertension may worsen postinfarct left ventricular remodelling [103, 104]. These conditions are associated with an increase in afterload, which contributes to hypertrophic remodelling of myocardial wall. It has been shown that replacement of a severely stenotic aortic valve and therapy to lower blood pressure in hypertensive patients could ameliorate concentric left ventricular hypertrophy [105, 106], but there are no data on postinfarct remodelling.

### 3.2. Biomarkers of Ventricular Remodelling

Cardiac magnetic resonance, which is the preferred imaging modality for the assessment of ventricular volumes and function, has been used to validate several putative biomarkers of ventricular remodelling. However, the clinical role of these biomarkers in predicting postinfarct remodelling needs further investigation.

A positive correlation has been found between ventricular remodelling and plasma levels of some enzymes that contribute to extracellular matrix remodelling, such as matrix metalloproteinases (MMP-2 and MMP-9) and tissue inhibitors of metalloproteinases (TIMPs) [58, 107].

Other plasma proteins whose levels positively correlate with ventricular remodelling are tissue plasminogen activator (t-PA) [58], a fibrinolytic enzyme that might play a causative role in postreperfusion intramyocardial hemorrhage [108] and that might contribute to extracellular matrix remodelling [109], terminal peptides derived from procollagen [58], some markers of systemic inflammation such as interleukin-1*β* and C-reactive protein (CRP) [58, 110], and some growth factors, such as hepatocyte growth factor (HGF), and growth differentiation factor-15 (GDF-15) [58].

The atrial natriuretic peptide (ANP), the brain natriuretic peptide (BNP), and the N-terminal fragment of its precursor (NT-proBNP) are produced by cardiomyocytes, and their blood levels increase with increasing myocardial wall stretch. In addition to their well known prognostic value in patients with heart failure, high levels of natriuretic peptides or NT-proBNP after myocardial infarction predict an increase in ventricular volumes, which is postinfarct remodelling [58, 108].

## 4. Consequences of Postinfarct Remodelling

Parameters that define left ventricular remodelling are consolidated surrogate end points [10]. In a meta-analysis, therapies that reduce end-systolic and end-diastolic volume, or that increase left ventricular ejection fraction, improve survival of patients. The use of surrogate end points in clinical trials is often advantageous, because it may render necessary fewer patients to demonstrate a statistically significant efficacy of one treatment. However, conclusions in clinical trials should never be based only on surrogate end points, but also on clinical events such as death or hospitalization for heart failure.

When a patient develops postinfarct left ventricular remodelling, he is at increased risk of heart failure or sudden death due to a lethal arrhythmia [11]. The qualitative alteration of left ventricular geometry and myocardial structure, together with increased fibrosis, predispose to anomalies in potential conduction that can result in reentrant arrhythmias [111]. Furthermore, the cellular changes that supervene during remodelling might increase the electrical automatism of ventricular myocytes [112]. Calcium overload in the cytosol is a trigger for arrhythmias sustained by afterdepolarizations, which are anomalous depolarizations that follow the normal action potential of cardiomyocytes [113]. They are often referred to as triggered activity [113].

Eccentric hypertrophy, as that observed during post-infarct ventricular remodelling, is associated with a threefold increase in the risk of major adverse cardiac events, including death from cardiovascular causes, reinfarction, heart failure, stroke, and cardiac arrest (HR: 3.1; 95% CI: 1.9–4.8, *p* < 0.01) [11]. Ventricular end-diastolic and end-systolic volumes directly correlate with mortality and rate of hospitalization for heart failure: Solomon and coworkers reported a hazard ratio of 1.06 (95% CI: 1.02–1.11, *p* = 0.009) per 10 mL increase in end-diastolic volume and of 1.11 (95% CI: 1.04–1.19, *p* = 0.001) per 10 mL increase in end-systolic volume [70]. Heart failure that develops following postinfarct remodelling is characterized by a reduced ejection fraction, because the infarcted myocardium has a suboptimal systolic function. A 5-unit decrease in LVEF is associated with about 30% increase in the risk of death or hospitalization for heart failure (HR: 1.29; 95% CI: 1.14–1.49, *p* < 0.001) [70].

**Figure 3:**
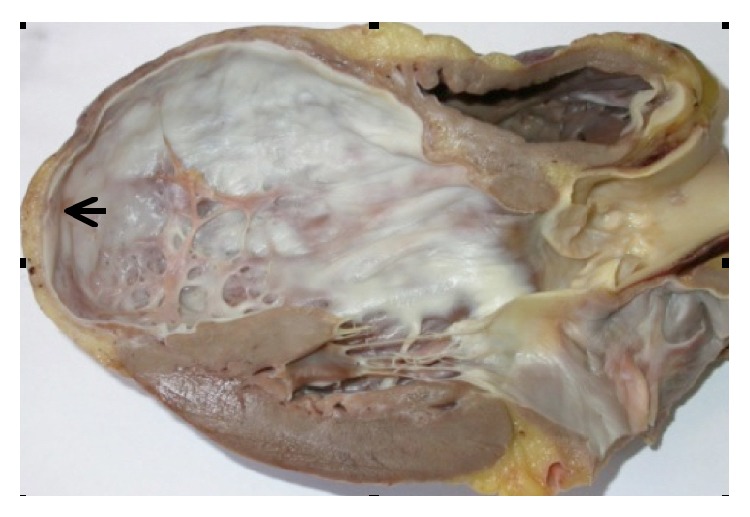
The expansion of a wide anterior and transmural infarct scar often leads to the formation of an apical left ventricular aneurysm that predisposes to left ventricular thrombosis. In this case, postinfarct remodelling is characterized by a great apical dilatation of the left ventricular chamber, together with a thinning of the infarcted segments (arrow).

Infarct scar expansion during postinfarct remodelling sometimes causes a great regional dilatation of ventricular chamber, which is a ventricular aneurysm (Figure 3). This process is usually observed with wide myocardial infarctions in the territory of the anterior interventricular artery. The myocardial wall of the ventricular aneurysm is constituted by the transmural infarct scar, with infarcted segments that are akinetic or dyskinetic [114].

The slow blood flow in the cavity of the ventricular aneurysm can lead to the formation of an intracardiac thrombus. Among 100 patients with an anterior ST segment elevation myocardial infarction and LVEF <40%, 27 patients had a left ventricular thrombus, as assessed by contrast-enhanced CMR [115]. A left ventricular thrombus is asymptomatic in the majority of cases, but it is associated with a low but significant risk of systemic thromboembolism (10–15% of cases), including strokes and transient ischemic attacks [114]. For this reason, patients with an intracardiac thrombus should be treated with anticoagulants [114].

## 5. Therapies against Postinfarct Remodelling

There are clinical evidences that postinfarct remodelling can be prevented or, in some cases, reversed [44]. This process has been termed reverse remodelling, and it could be accomplished with either pharmacologic or mechanical interventions, or with a combined approach. While mechanical approaches require surgery and are reserved to patients with symptomatic heart failure who meet strict eligibility criteria, drugs are the preferred strategy for treating patients with mild heart failure or for preventing postinfarct ventricular remodelling.  Table 2 summarizes the current therapies that are capable of inducing reverse remodelling, together with some of the experimental interventions that have been effective in pilot trials.

Angiotensin-converting enzyme inhibitors (ACEi) and angiotensin receptor blockers (ARBs) have consolidated efficacy as antiremodelling drugs [70, 71], because of their action as antagonists of the renin-angiotensin-aldosterone system (RAAS) that plays a major causative role in ventricular fibrosis. A combined therapy with an ACEi or an ARB and an antialdosterone diuretic is more effective than a monotherapy in reversing remodelling [71].

The recent PARADIGM-HF multicenter randomized controlled trial [116] tested a drug that is a combination of the ARB valsartan and the neprilysin inhibitor sacubitril on 8399 patients with heart failure and a reduced LVEF, reporting a reduction of 20% in death from cardiovascular causes or hospitalization for heart failure as compared with the ACE inhibitor enalapril alone at maximum dosage (*p* < 0.001). Neprilysin is and endopeptidase that degrades several vasoactive peptides, such as natriuretic peptides, bradykinin, and adrenomedullin [116]. It is likely that increasing all these substances in blood through neprilysin inhibition protects the heart from remodelling, in particular when it is associated with the inhibition of the RAAS. It is desirable that a meta-analysis of large clinical trials confirms the efficacy and safety of this new combined approach, before it enters clinical practice in the management of heart failure.

Together with ACEi, ARBs and antialdosterone diuretics, *β*-blockers are the current mainstay of pharmacologic therapy against postinfarct remodelling [73].

Adrenergic stimulation allows for the maintenance of an adequate global cardiac function after acute myocardial infarction, by increasing the contractility of viable myocardium. However, chronic *β*-adrenergic overstimulation has cardiotoxic effects, leading to left ventricular dilatation and systolic dysfunction [68, 69]. *β*-blockers might improve autonomic control of failing heart by increasing the number of *β*-receptors on cardiomyocytes and by modulating their activity [44, 117]. Apart from its beneficial effects on remodelling, a long-term therapy with *β*-blockers reduces mortality after an acute myocardial infarction, by reducing the risk of a lethal arrhythmia [118].

Nitric oxide (NO) donors such as nitrates have well known beneficial effects in patients with heart failure [44, 74] and might induce reverse remodelling by reducing the preload, as well as increasing cyclic guanosine monophosphate (cGMP) in cardiomyocytes [44, 119]. cGMP protects cardiac cells from apoptosis [44, 119, 120].

As the major risk factor for postinfarct remodelling is infarct size [20], therapies against ischemia-reperfusion injury during acute coronary syndromes should be expected to prevent postinfarct remodelling. As a proof of concept, experimental therapies that improve myocardial salvage during PPCI, such as cyclosporine and ischemic postconditioning, have been associated with maintenance of left ventricular volumes, but larger confirmatory trials are required [121].

Other promising approaches are stem cells and gene therapy, which have shown interesting results in pilot trials on adjunctive therapy of myocardial infarction and heart failure, and that might reverse postinfarct remodelling [5, 122].

## 6. Conclusions

Therapies with proven efficacy against postinfarct remodelling exist, and research is bringing new discoveries in the pathogenesis of postinfarct remodelling into the field of clinical practice and therapy. Heart failure is one of the most important causes of morbidity and mortality worldwide, and patients with postinfarct remodelling show the highest risk of symptomatic heart failure. For this reason, the battle of medicine against heart failure is against postinfarct remodelling, which means that the prevention is better than the cure.

## Figures and Tables

**Table 1:** Molecular pathways of ventricular remodelling. Many mediators have either an adaptive role (in bold) at low doses or a maladaptive role, with chronic/intense stimulation.

	Molecular pathways activated by interaction with receptor	Effects on cardiomyocytes
*Soluble mediator*		
Angiotensin II [28]	JNK **ERK**	Apoptosis Cell survival and growth
ROS [28, 60]	Cell damage **JAK/STAT**	Apoptosis Cell survival and growth
TNF-*α* [28, 61, 62]	NF-*κ*B **NF-*κ*B**	Apoptosis Cell survival and growth
Growth factors (IGF-1, PDGF, GDF-15, HGF, and NRG-1) [28, 59, 63, 64]	**PI3K/AKT** **RAS/RAF/MEK/ERK**	Cell survival and growth
Cardiotrophin-1 [28, 65]	**JAK/STAT**	Cell survival and growth
Cytosolic calcium [59, 66, 67]	Calpains (calcium-activated proteases) **Calcineurin/NFAT**	Apoptosis Cell survival and growth
Catecholamines [68, 69] (*β*-adrenergic signalling)	PKA **ERK**	Apoptosis Cell survival and growth

*Mechanical sensing of myocardial stretch*		
Integrins [54]	**FAK**	Cell survival and growth

JNK: Jun N-terminal kinase; ERK: extracellular-regulated kinase; JAK/STAT: Janus kinase/signal transducers and activators of transcription; ROS: reactive oxygen species; TNF-*α*: tumor necrosis factor-*α*; TRADD: TNF receptor-associated death domain; NF-*κ*B: nuclear factor-*κ*B; IGF-1: insulin-like growth factor-1; PDGF: platelet-derived growth factor; GDF-15: growth differentiation factor-15; HGF: hepatocyte growth factor; NRG-1: neuregulin-1; PI3K/AKT: phosphatidylinositol 3-kinase/AKT; NFAT: nuclear factor of activated T cells; PKA: protein kinase A; FAK: focal adhesion kinase.

**Table 2:** Therapies capable of inducing reverse remodelling.

	Mechanism of action	Notes
*Class of drugs*		
ACE inhibitors/ARBs [70, 71]	RAAS antagonism	
Antialdosterone diuretics [72]	RAAS antagonism	
*β*-blockers [73]	Reduce cardiotoxic effects of chronic *β*-adrenergic stimulation and improve heart responsiveness to physiological adrenergic stimulation	
NO donors plus hydralazine [74]	Increase cGMP and reduce preload	
MMPs inhibitors [75]	Inhibit ECM remodelling	Experimental. No evidences in humans
rNRG-1 [76, 77]	Promotes cardiomyocyte survival pathways	Experimental

*Type of mechanical intervention*		
CRT [44, 78]	Increases GSK-3*β* activity and improves LV contractility	Eligibility: patients with symptomatic HF and LBBB
LVAD [44, 79]	Reduces LV workload	Eligibility: patients with severe HF as bridge to recovery or bridge to heart transplant
Mitral valve surgery [80]	Reduces LV workload	Eligibility: patients with severe mitral regurgitation
Diastolic cardiac restraint devices [81, 82]	Reduce myocardial wall tension	Experimental

ACE: angiotensin-converting enzyme; ARBs: angiotensin receptor blockers; RAAS: renin-angiotensin-aldosterone system; NO nitric oxide; cGMP: cyclic guanosine monophosphate; MMPs: matrix metalloproteinases; ECM: extracellular matrix; rNRG-1: recombinant human neuregulin-1; CRT: cardiac resynchronization therapy; GSK-3*β*: glycogen synthase kinase-3*β*; LV: left ventricle; HF: heart failure; LBBB: left bundle branch block; LVAD: left ventricular assist device.
